# Supplementary figures and images for: Deep evaluation of the evolutionary history of the Heat Shock Factor (HSF) gene family and its expansion pattern in seed plants
Source: PeerJ. 2022 Aug 9;10:e13603. doi: 10.7717/peerj.13603 (PMC9373977; doi:10.7717/peerj.13603)

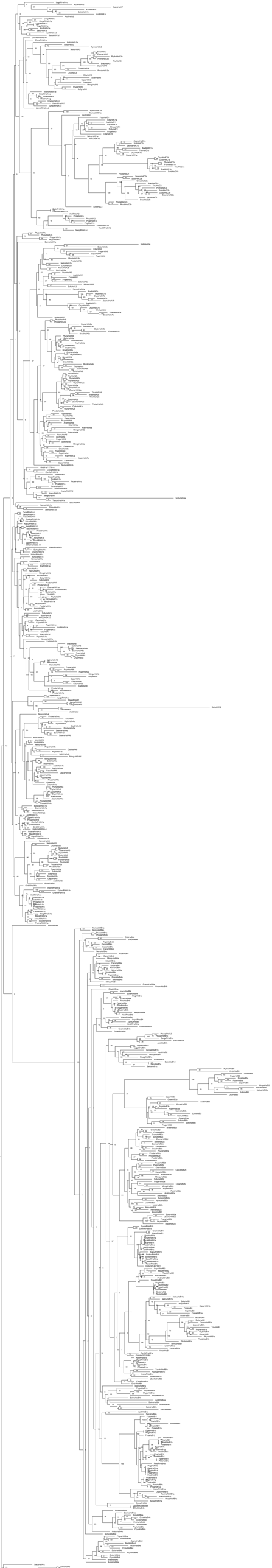

Supplement: Figure S1 — The classifications are based on HSFs annotations identified using the HEATSTER site (https://applbio.biologie.uni-frankfurt.de/HSF/heatster/). [file peerj-10-13603-s003.pdf]

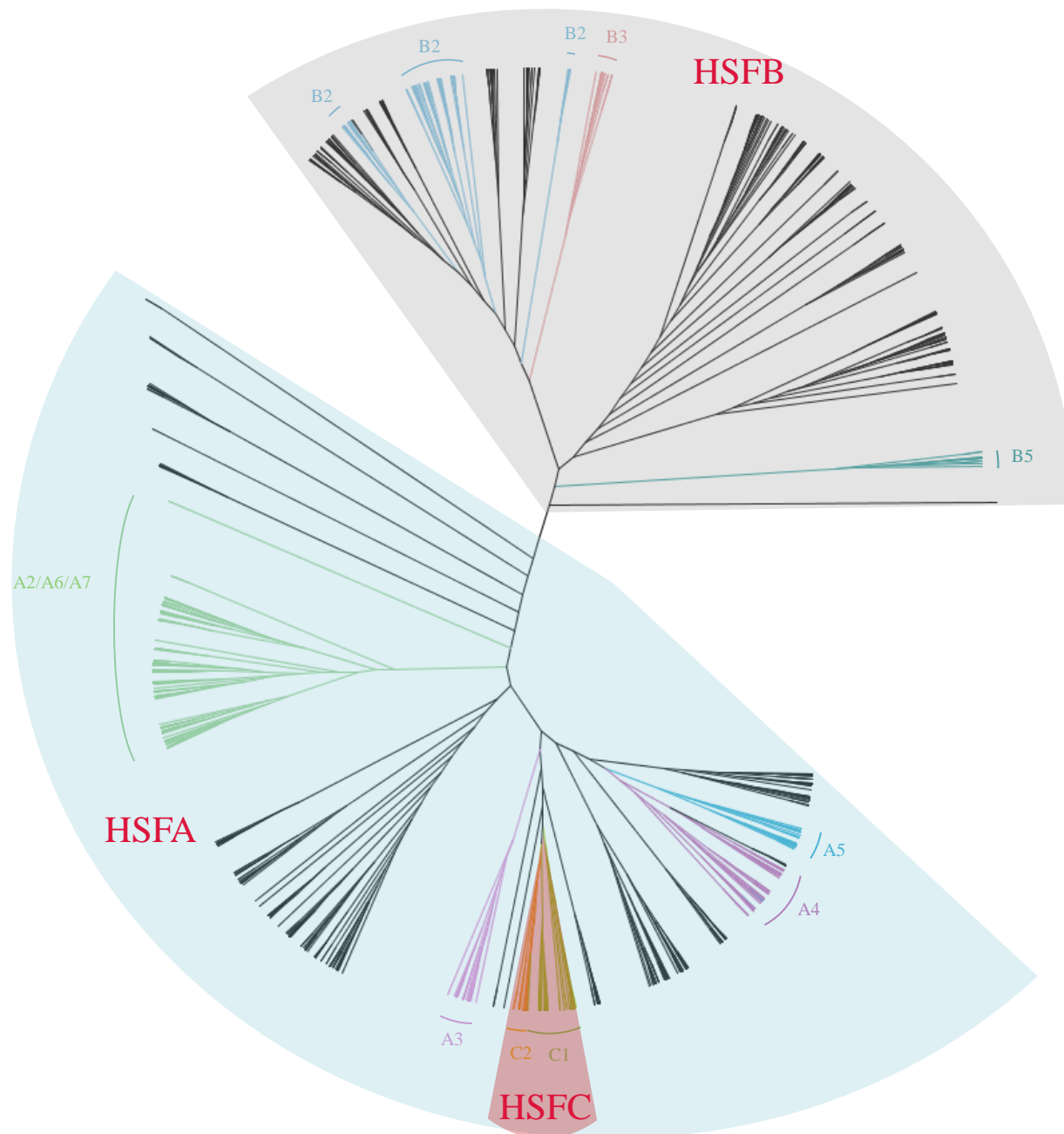

Supplement: Figure S4 — HSFA, HSFB and HSFC are clustered into three main clades. The clade of subfamilies HSFA2/A6/A7, HSFA 3, HSFA 4, HSFA 5, HSFB2, HSFB3, HSFB5, HSFC1 and HSFC2, were shown over relevant branches with different colors. The three groups HSFA, HSFB, and HSFC were highlighted with shades of different colors. [file peerj-10-13603-s006.pdf]

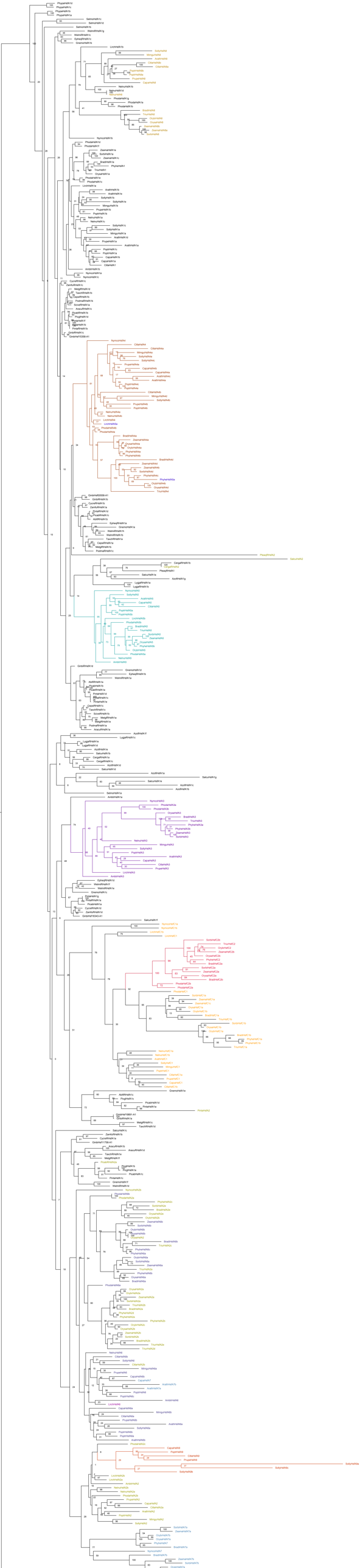

Supplement: Figure S5 — The branch and the genes’ name of distinct clusters of HSFA3, HSFA4, HSFA5, HSFA8, and HSFA9 were color coded. In the others, only the gene names were colored. The relevant support values are shown. The scale bar represents amino acid substitutions per site. [file peerj-10-13603-s007.pdf]

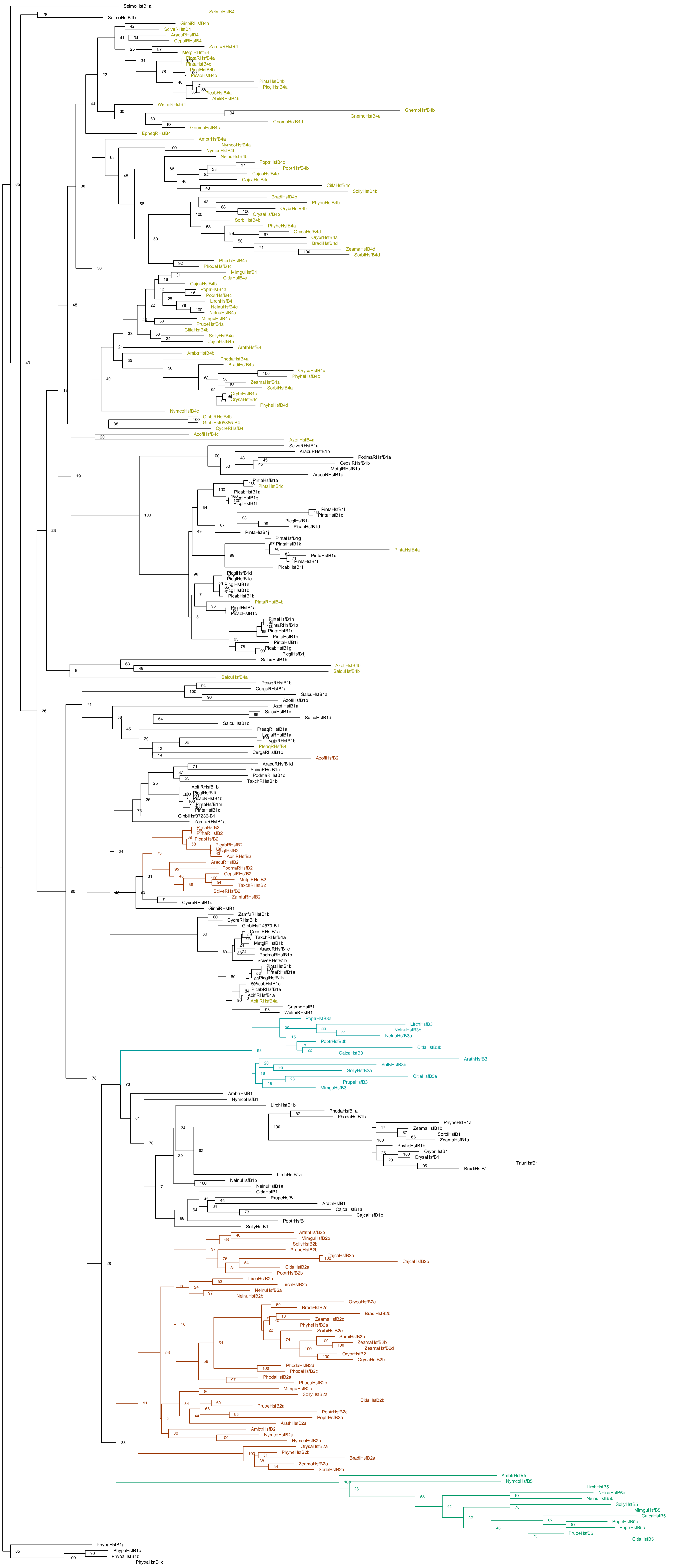

Supplement: Figure S6 — The branch and the genes’ name of distinct clusters of HSFB2, HSFB3, HSFB5 were color coded. In the others, only the gene names were colored. The relevant support values are shown. The scale bar represents amino acid substitutions per site. [file peerj-10-13603-s008.pdf]
