# Supplementary material for: Deep evaluation of the evolutionary history of the Heat Shock Factor (HSF) gene family and its expansion pattern in seed plants
Source: PeerJ. 2022 Aug 9;10:e13603. doi: 10.7717/peerj.13603 (PMC9373977; doi:10.7717/peerj.13603)

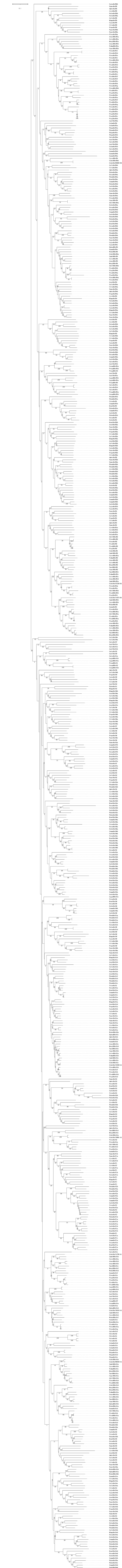

Supplement: Figure S3 — The bootstrap values were showed on tree. [file peerj-10-13603-s005.pdf]
